# Book Reviews

**Published:** 1814-08

**Authors:** 


### CRITICAL ANALYSIS

### *OF RECENT PUBLICATIONS*

IN THE

### DIFFERENT BRANCHES OF PHYSIC, SURGERY, AND MEDICAL PHILOSOPHY.

*A Treatise on the Hereditary Properties of Diseases; containing Remarks on the unfounded Terrors and ill-judged Cautions consequent on such Opinions: with Notes, illustrative of the Subject, particularly in Scrofula and Madness.* By JOSEPH ADAMS, M.D. F.L.S. Physician to the Small-pox Hospital, &c. 8vo. boards. 5s. 6d.

HEREDITARY diseases are a fruitful source of terror to the patient, and of inquiry to the physician. One should therefore have expected that before this time the subject would have been generally understood, or at least accurately canvassed. Yet, if we may believe our author, no systematic inquiry on the subject has been instituted from the days of Mercatus, who wrote at the beginning of the seventeenth century, till our own times, a period of nearly two hundred years. When the Memoirs of the French National Institute presented us with M. Portal's paper,* on account of the celebrity of the author, as well as the novelty of the inquiry, we thought it right to make our readers acquainted with that essay; and, if its appearance has given rise to the present performance, we shall think our time and labour amply repaid.

The work commences with a distinction between hereditary and family diseases; the former are such as are transmitted from parents to their offspring, the latter such as appear in more than one of the offspring of parents free from such complaints. These distinctions are carefully kept in view throughout, and by attending to them an inference is

* For a translation of M. Portal's paper, extracted from the French National Institute, see Med. and Phys. Journal, vol. xxxi. p. 229, and 281.

drawn drawn, that connate diseases or privations of senses are rarely, if ever, hereditary, but often appear among brothers and sisters, the offspring of parents free from such inconveniences.

The different periods of life at which such diseases show themselves, forms the next division; and on these the prognosis of the access, and even cure of such complaints, are made very much to depend. As the deductions are drawn from a great number of observations, collected with much industry, we shall not offer any opinion upon them, but in our short analysis at once introduce our readers to the last and most important part of the inquiry, namely, "what provision is made by Nature to correct the influence of such hereditary causes, and how far they can be imitated and improved by art?" These provisions are pointed out in the influence produced by climate, in the result of customs during the less cultivated state of society, and also in the progress of refinement; and lastly, in the positive laws, human and divine, which seem to have had such a provision in view. That these provisions are sufficient, in almost every case, is shown by the present state of mankind, and by the necessary consequence that would have followed the want of them. This is most strikingly illustrated in a single instance, in which none of these could avail, because the cause would be constantly operating; and here a special provision is made by an effect of the disease itself, which entirely supersedes propagation from such sources.

"But should there exist a disease, (says our author,) the disposition to which is excited by climate; should such a disposition become hereditary, and should the disease when excited prove incurable, from such a combination of causes we could expect nothing less, than the gradual extinction of the race; and should the district be repeopled, the same succession of causes and effects must gradually extinguish the descendants of the new Colonists; yet, such a disease does exist in the finest and most extensive part of the habitable globe. Human institutions have indeed made some feeble attempt at restraining it, but human endeavours must have proved ineffectual. Happily, the same power which permitted such a cause, has fixed limits to its effects.

"The *Elephantiasis* of Aretæus is peculiar to warm climates; the disposition to the disease is hereditary, and the disease itself has, hitherto, proved incurable. I have never been able to learn, that it has attacked emigrants from a colder climate, nor their immediate descendants. A residence therefore of some generations, is probably necessary to induce the disposition. When this diseased disposition is *derived* from *inheritance*, the action always commences before the age of puberty; and the subject never arrives at that state; the organs are never evolved, and no other marks of virility appear. When the the disease originates with an individual, it usually commences at a more advanced age; but from that time, the organs which distinguish the sexes decay, and become gradually unfit for their original purposes. The fact of a disease, which arrests the progress to virility of every youth, and emasculates every adult whom it attacks, is so surprising, that after having witnessed it myself, I should have been backward in publishing the result of my observations, had not others been present at every examination; and I should have been unwilling to draw inferences from them, had not subsequent writers confirmed my account.*

"Thus an hereditary disposition to an irregularity of the most formidable nature, which being excited by climate, must have progressively increased in spite of all human institutions, is arrested as soon as it occurs, by those very actions which form a part of the deviation from the usual progress of Nature."

Such is the short analysis our limits will afford of this interesting work. A great number of notes are added, which may be said to form distinct essays. We shall particularly notice one as connected with that peculiar property of Elephantiasis, to which our extract refers. The author seems fearful lest a passage in Dr. Bateman's Synopsis should seem to invalidate his testimony on a fact to which he attaches so important a final intention. Without entering into the controversy we shall briefly state its outlines. Aretæus is the first writer from whom we can collect any accurate description of Elephantiasis. Among the symptoms, he mentions *libido inexplebilis*, in which he has been followed or at least uncontradicted by every subsequent author till Dr. Adams. We need not say how much the symptom described by him is at variance with the salacious temper imputed to these unhappy objects, nor need we enter into any speculations on its existence after the notice given in our former Number of the case now in St. Bartholomew's Hospital, which any of our readers may examine.

The passage concerning the Itch, its true diagnosis, and the various eruptions confounded under the same name, is not the least interesting, nor by any means the least important part of the work; whether we consider the frequency of the disease, or how little it is understood by those whose practice is confined to the higher ranks. It also gives Dr. Adams an opportunity of descanting on one part of the study of medicine, the influence of which is become so extensive,

* "See Edinburgh Medical Journal, vol. v. p. 500, Note.—Gentleman's Magazine, vol. lxxxi. July to December, 1811, p. 145, second column;—and Dr. Gourlay's History of Madeira."

4 that, that, if ill directed, it must prove highly injurious to mankind, in a science so necessary to its comfort as the treatment of disease; for it cannot be necessary to add, that the slightest misnomer may introduce a fatal error into practice. If we understand our author, this is the object he has in view in objecting to the word TYPHUS, which he seems to hint has been the source of an error in the army practice, which is now pretty generally admitted. The passage is so pointed that we shall make no apology for transcribing it.

"These remarks on names, somewhat abruptly introduced, may serve to show how careful we should be in changing commonly-received terms. Whether we use psora or scabies for itch, may seem of little consequence, as it is not certain that those from whom we derive the words were acquainted with the disease to which we now apply them. But whenever a name is changed, some regard should be paid to etymology, as the great use of nosology is, that we should all be acquainted with each other's language. If we expect more than this, there will be great danger of misleading ourselves. The first person who proposed an arrangement of diseases, similar to what is made of plants, was Sydenham. He, however, rather looked to it with a wish than an expectation of its accomplishment, and seems not aware that the arrangements of botanists were, in many instances, artificial. It is certain, that he lived to lament the application of practice to names, declaring, in the most advanced period of his practice, that 'the invention of the term, or opinion of malignity, had been far more destructive to mankind than the invention of gunpowder.' I leave it to the decision of those who have had most experience, in the comparative effects of disease and gunpowder, how far the introduction of the term Typhus may be liable to a similar charge. We may at least remark, that the very fever which drew this expression from Sydenham, is, by Dr. Cullen, included among the Synonyma of Typhus.* As the illustrious Professor refers to every original author, it would be unreasonable to accuse *him*, if such sources of information are not studied by his readers; and if he made his Nosology a text-book for his lectures, we cannot doubt that his hearers were often apprised, that the same mode of practice could not be applicable to fevers arising from so many causes, assuming so many forms, appearing in such different climates, and under habits of life so different as to comprehend near fifty synonyms. Still, we may lament the influence of a term, in a work which, from the just celebrity of the writer, and its connection with the 'First Lines,' may hereafter become the text-book of less enlightened lecturers, as it is already of physical compendiums.

"Diseases of the skin being more immediately the objects of our senses, may be thought more easily reducible to orders and genera. Let us try this in Itch and Syphilis; because, in these we are most frequently required to give a decided opinion. Of the first, if

* "Febris nova anni, 1685, Sydcn. Scedul. Monitoria.

enough enough has not been said, to show the difficulty which attends such an attempt, we may add from the Synopsis, that 'from its affinity with three orders of eruptive appearances, pustules, vesicles, and papulæ, it *almost* bids defiance to an artificial classification.' The above orders, comprehending only fifteen genera with their species and varieties, may be less alarming to a modest enquirer. But the eruptions of Syphilis are said to 'bid defiance to arrangement, according to external character;' that, 'in fact, they possess no common or exclusive marks, by which their nature and origin are indicated;' and that 'there is no order of cutaneous appearances, and scarcely any genus or species of chronic eruption *already described*, [and this is the last,] which these secondary symptoms of Syphilis do not occasionally imitate.'—'Nevertheless,' continues Dr. Bateman, 'there is in many, a difference which a practised eye will recognize between the ordinary diseases of the skin and the syphilitic eruptions to which the same *generic appellation* might be given; this is often observable in the shade of colour, in the situation occupied by the eruption, in the mode of its distribution, and in the general complexion of the patient. Hence, to a person conversant with those ordinary diseases, a degree of anomaly, in these respects, will immediately excite a suspicion, which will lead him to investigate the history of the progress of such an eruption, and of its concomitant symptoms. And it will frequently happen, that the most experienced observer can only arrive at a satisfactory conclusion, by comparing the cutaneous appearances with these concurring symptoms, and with the previous history of the disease.' [Synopsis, p. 328.] Fortunate are those tyros and practitioners who are within the reach of consulting a person conversant with those ordinary diseases, as the discriminating marks between half a score such and syphilis, can afford little assistance to the common reader.

"This Note may seem extended to an unreasonable length, and no longer connected with hereditary diseases. But it is most intimately connected with whatever relates to medicine as a science. For if two not uncommon diseases, the progress of which is slow enough to admit the most accurate observation, are reduceable to no laws, or to none that can be described, it is evident that our present mode of investigation must be defective, or that we must give up every prospect of progressive improvement. We are told, from very high authority, that the faculty of tracing actions, so as to discover a law, makes the whole of philosophy.* In medicine this must be more difficult, and more as well as closer observations must be necessary, than in any other branch of natural knowledge; because diseases are modified by constitutions, and their progress interrupted by remedies. Hence, it is not easy to bring men to a general agreement; yet, some progress has been made towards such an event. In the Small-pox, Sydenham has taught us to look for certain laws, which were never established till his time.† In gout, he has not been less successful; and the instructions he has given in tracing fevers,

"* Nov. Organ." "† See Mead's Discourse, chap. 2."

have have become almost oracular. Fothergill has been scarcely less successful in Scarlatina. Though in the treatment of these diseases, improvements may be expected, and changes necessary under different circumstances, yet the phenomena of the diseases themselves, as marked by those writers, are now so familiar to us all, as to admit of no dispute. If this can be accomplished in fevers, shall we despair of arriving at the same certainty in diseases, whose progress is slower? It must be admitted, that for so desirable an event, we require the same talent at observation, the same persevering diligence, with the same integrity as existed in the man who, after establishing such laws, should declare that there are small-pox cases, which the errors of a nurse could not render fatal; and others, which the skill of no physician could cure. When, therefore, we possess the fruits of such men's labours, let us learn how to value them.

"Having made these general remarks, I shall trace very shortly the progress of Mr. Hunter's inquiries, in a disease which is said 'to possess no common or exclusive marks.'"

Dr. Adams proceeds to trace the very confused history given of venereal complaints before Mr. Hunter's time, and the greater accuracy which his discrimination has introduced. This he proves in a most striking manner by the controversy which at one time existed as to the importation of that disease into Otaheite, two nations mutually accusing each other as the authors of an event which there is reason to believe never existed. We should dwell longer on this and several other passages, were it not that from what we have already produced, and from the well-known character of the author, we cannot doubt that the work will be in the possession of most of our readers.

---

*Medico-Chirurgical Transactions, published by the Medical and Chirurgical Society of London. Volume the Fourth.* 8vo. pp. 490.—Longman and Co. 1813

(Continued from p. 74.)

XIII. *History of a Case of Premature Puberty.* Communicated by ASTLEY COOPER, Esq. F.R.S.

THESE rare instances of precarious developement of sexual organs and preternatural growth, are curious, though at present we are content with noticing them, without attempting to account for their existence.

"Charlotte Mawer, the daughter of a waterman at Lincoln, aged about four years and a half, had an appearance of the catamenia about a year and a half since, and again in the course of four or five months; but for the last two or three periods, nearly at the end of five weeks. The discharge exactly resembled that of most women, except that it was of rather a darker colour. The last period was about the 5th of March, 1813, when the girl looked pale, and seemed to have a degree of lassitude about her. The breasts are very full, and as large as most young women's of twenty years of age. She is very broad over her chest and loins. Her pelvis seems much larger than is ever observed at her age. The pubes is covered with a white-coloured hair, which began to shew itself when she had the first appearance of the catamenia. She is quite a little woman in her appearance, except as to her countenance, which is childish. She is much bigger than a sister who is two years older. She plays with children of her own age, and does not seem to have any sexual feelings, or an uncommon degree of modesty. That there might be no mistake with respect to her age, the register of the parish where she was baptised was examined, which specified her being born on the 22d of March, 1806.

"Since the above account of the case of Charlotte Mawer was taken, she has continued to menstruate regularly; but there is now only a space of three weeks between each period. The discharge is as copious as in most women, and it generally continues about four days; it is the colour of venous blood, and does not coagulate. She is frequently affected with the leucorrhœa in the intervals; the hair has begun to grow in the axillæ; her countenance has not near so much the childish appearance; her voice is much rougher; she has a degree of modesty not formerly noticed, and does not now like to walk in the streets, because some boys have teazed her about her appearance. She is four feet and an inch high, is broader across the pelvis than the shoulders, measuring only fourteen inches and three-quarters from one acromion scapulæ to the other, but seventeen inches from the anterior superior spinous process of one ilium to that of the other.

"Her eldest sister, aged about seventeen years, has never menstruated, and there is very little fullness in her breasts, though she is in good health. One of her sisters, aged ten years and five months, is four feet and two inches high, and is not so broad across her pelvis as her shoulders; she measures fourteen inches and a half from one acromion scapulæ to the other, and thirteen inches from the anterior superior spinous process of one ilium to that of the other."

A somewhat similar instance in a girl, is recorded in the 17th volume of this Journal, p. 522; and an ingenious paper on the subject, is inserted in our 10th volume.

XIV. *Case of Contracted Wrist, successfully treated.* By Mr. HODGSON, Surgeon, at Lewes.

THE subject of this case was a lady aged twenty years, in whom "the carpal bones were so completely dislocated, that their posterior part, with the ends of the radius and ulnæ, represented the anterior part of a stump: the fingers were strongly clenched into the palm of the hand, with the knuckles resting upon the inside of the fore-arm, opposite the flexor tendons." The origin of this affection was attributed to convulsion fits. Mr. Hodgson succeeded in restoring the the parts to their natural condition by the following means. Having immersed the hand in warm water, he succeeded in getting the little finger sufficiently out to admit of a small bolster of wool wrapped in linen. This was allowed to remain within the grasp of the finger: the other fingers were successively treated in a similar manner, the size of the bolster being gradually increased. At the same time Mr. H. endeavoured to bring the hand back; "but (he observes) the rigidity of the wrist was so great, that had not my patient possessed an uncommon share of courage, she must have been disheartened from any further attempt. By the use, however, of warm water, and embrocating the parts with oil, it gradually gave way, in the course of about ten weeks, to a moderate degree of force applied by my hand daily." The author derived advantage from an apparatus which he contrived of a spring made of iron, which projected from the back part of the wrist over the back of the hand. To this a piece of soft leather, passed round the inside of the fingers and palm of the hand, was fixed, and thus the hand was effectually kept in its situation.

XV. *History and Dissection of a fatal Case of Cynanche Laryngea.* By Edward Percival, M.D. Dublin.

This case is so highly interesting, both from the style in which it is narrated, the peculiar circumstances which attended it, its termination, and the scope which it affords for reflection, that we shall present it to our readers as communicated by the learned author.

"The following recital of a case of cynanche laryngea, whose fatal event has lately deprived the world of an eminent character, is transcribed from notes taken daily during my personal attendance upon him, in conjunction with another physician and two surgeons.

"X. Y., aged 63, of spare, though muscular habit, and somewhat delicate health, was attacked with apparently slight catarrh and sore throat, in consequence of a hasty and fatiguing journey. On Tuesday, May the 4th, 1813, he consulted an eminent physician of this city, who advised purgative and sudorific remedies, under which treatment the symptoms appeared wholly to subside. Having imprudently ridden out, and exposed himself to a cold east wind, the catarrhal affection recurred on Friday the 7th, when he was directed to be bled to the amount of 16 ounces, to take a pill of calomel and cathartic extract, and a draught with nitre. He rested perfectly well this night, and appeared nearly in his usual health on the following morning. His tongue was clean, his pulse between 70 and 80 strokes in the minute; and he took an airing in his carriage in the course of the day. On the following morning (Monday) he again manifested catarrhal symptoms, with sore throat, and hoarseness. He was now directed to take James's powder to some extent, which induced profuse perspiration, with great relief of all the urgent symptoms. Before the sweating had subsided, on Tuesday morning, he injudiciously rose from his bed, and occupied himself during the day, by dictating aloud to some attornies in his chamber. In the evening, his respiration became much impeded, and his voice extremely hoarse; which alarmed him by the resemblance of the present attack, to one which he experienced in London, eleven years before. On that occasion, he was bled copiously and repeatedly, and narrowly preserved his life. The patient himself, and several of his friends, affirmed the disorder to be the same as the present, and confined to the upper parts of the windpipe. It may be proper to observe, that his father suffered a severe attack of this kind many years ago.

"On Wednesday morning, I saw him for the first time, in conjunction with another physician and a surgeon. His countenance was then pale and anxious; his eyes protruding; his tongue foul and much swelled; his respiration slow and laborious, with a shrill or stridulous sound, as of air forcibly passing through a narrow orifice; and his voice indistinctly audible, in the tone of a hoarse whisper. He had a perpetual inclination to expectorate; but all his efforts to this purpose were fruitless, which added much to his distress, by the continual apprehension of suffocation. He was perfectly unable to swallow any substance, whether fluid or solid, as the smallest portions of either were instantly rejected with violent coughing. The visible internal fauces were of natural appearance and pale colour. He informed us that he had experienced some shooting pains about the larynx, which were now entirely subsided. The epiglottis was swollen or distended, and somewhat erect; which accounted for the coughing and sense of suffocation on attempting to swallow any-thing, as this organ had ceased to act as a valve upon the larynx. His pulse was full, throbbing, and very frequent.

"Twenty ounces of blood were withdrawn from his arm by a large orifice, which afforded some immediate relief to his respiration, and his general feelings of distress. He now thought he was able to swallow, but, on attempting it with a tea-spoonful of water, the fluid was rejected with a fit of coughing, or rather of suffocation, which nearly extinguished life.

"Sixteen leeches were now applied to his throat, and two purgative enemata were administered, which brought away no fæces.

"In the afternoon, his urgent symptoms appeared to be stationary, twelve ounces of blood were taken from his arm, and a dozen leeches applied to his throat.

"At nine o'clock, p. m. his pulse was softer and less frequent, but his tongue was still swelled and dry, his breathing not amended, and the stridulous sound unabated. Bronchotomy was now determined upon; and the operation was performed without delay, by a vertical division of the integuments covering the interval between the cricoid and thyroid cartilages; the tracheal membrane was pierced laterally, and a canula was inserted, whose diameter was somewhat less than half an inch. The loss of blood by this operation might be about about six ounces; some part of which escaped into the trachea, and was returned through the aperture with much gurgling noise. He likewise expectorated a small quantity of mucus. In about half an hour, he breathed with perfect facility, and fully inflated his lungs, which was hailed as a favourable circumstance. He slept at intervals during the night, for the space of three hours; and towards the morning, he swallowed four dessert spoonfuls of milk, with some caution and address.

"At half past ten o'clock, a. m. (Thursday) his pulse was frequent but tranquil, his animal spirits lively, and he wrote much with a pencil upon paper, concerning various matters. He spoke with effort, and the tone of his voice was still that of a hoarse whisper. Two or three purgative injections brought away a trifling quantity of fæcal matter. At noon, he made a fruitless attempt to swallow. A nutritive injection of milk, and another of broth, were administered in the course of the afternoon and evening. Eight leeches also were applied to his throat. During the night, his pulse became more frequent, and he continued, though restless, to lie on his back only when recumbent. By great, and repeated efforts, he expectorated a piece of hardened mucus besmeared with blood. About two o'clock in the morning, his breathing became greatly embarrassed, his extremities grew cold, and he appeared to be on the point of death. Twelve ounces of blood were withdrawn from his arm; the canula was cleansed and replaced (an operation which was always repeated at short intervals) when to the agreeable surprise of his attendants, his respiration became more easy, the warmth returned to his extremities, his pulse revived, and shortly afterwards his animal spirits and cheerfulness returned. He swallowed a pint of liquid nourishment, at intervals, with tolerable facility; and he repeatedly expectorated pieces of hardened mucus besmeared with blood.

"At ten o'clock, a. m. on this day (Friday) he appeared refreshed and amended in all respects. Purgative and nutritive enemata were administered during the day alternately. He swallowed also small portions of broth and jelly repeatedly. In the evening, his pulse was soft and tranquil, though still frequent. His supine posture was, for the first time, changed to lying on his right side. His powers of deglutition were considerably improved, and the tone of his voice greatly amended. He had passed fæces and urine. His tongue, which had hitherto been swollen and hard, was now reduced to little more than the natural size, and was soft and moist; his countenance also was much improved; but he continued to breathe only through the canula. He passed the early part of this night tranquilly, until two or three o'clock in the morning, when his fever increased (similarly to the exacerbation of the preceding night), his breathing became laborious, and he evinced much irritability and despondency of mind. This febrile paroxysm continued for several hours.

"At noon on Saturday, his symptoms again wore a favourable appearance. His countenance was natural; his pulse, though frequent, was regular and firm; his tongue was reduced to its natural size and appearance; his powers of deglutition were perfectly restored; and the tone of his voice approached to a low key of the natural sound. He had passed copious evacuations, by a solution of Rochelle salt in chickenchicken-broth. The canula had been removed from the trachea for some hours; and as he breathed with perfect facility through the natural organs, it was not afterwards inserted into the aperture.

"The evening, and early part of this night, were passed with tranquillity and some sleep. About sunrise, however, he began to shew much impatience, irritability, and alarm. Yet he swallowed, at six o'clock, a. m. on Sunday morning, six or seven spoonfuls of oatmeal porridge, with milk, at his own particular request. About two hours afterwards, his mind appeared to be much disturbed; his pulse became more frequent and feeble; delirium, and at length stupor supervened, and he sunk rapidly until six o'clock in the afternoon, when he died without a struggle.

"At three o'clock on the following day, one of the surgeons who had been in attendance, (and who is a skilful professor of anatomy in this city,) opened the thorax and examined the larynx in my presence. The following is an account of the appearances dictated at the time of the dissection.

"On opening the thorax, the cartilages of the ribs proved to be ossified. The left side of the mediastinum, and the surface of the lung contiguous to it, appeared quite dry, as though the parts had been long exposed to air. A similar appearance, though in a less remarkable degree, manifested itself on the right side. The lungs were perfectly sound and natural, excepting a slight adhesion of the right lung to the costal pleura. Somewhat more than half an ounce of aqua pericardii was discovered; the heart itself was perfectly natural.

"The membrane common to the larynx and œsophagus, was very much thickened. The epiglottis was perfectly natural; but from the sides of this, to the arytenoid cartilages, the parts were morbidly thickened, increasing in density, as they approached the cricoid cartilage. The orifice of the rima glottidis was somewhat diminished, but exhibited no appearances of unusual vascularity. On dividing the membranes in the posterior part of the trachea, its internal surface appeared coated with thin pus, which continued to shew itself to the base of the cricoid cartilage, and between the membrane common to the œsophagus and larynx, and the posterior surface of the cricoid cartilage. Passing a probe into this part, it was discovered that between this membrane and the muscles of the larynx, even to the points of the arytenoid cartilages, was formed one extensive abscess.

"The upper edge of the cricoid cartilage was ossified, and found bare and rough by loss of surface as from exfoliation. The right arytenoid cartilage was dislocated, and when moved on the cricoid, gave the sensation of two bones, stripped of their articulating cartilage, rubbing together.

"Tracing the course of the abscess just mentioned, along the right side of the larynx, it was found to extend between the cricoid and thyroid cartilages, until it reached nearly to the edge of the artificial opening made during life, without however communicating with it in any part.

"*Remarks.*—On reviewing the history of the preceding case, the first first circumstance of remark is the unexpected suddenness and violence of the morbid affection of the larynx; an affection which has been so ably and discriminately defined by Dr. Farre, in his valuable communications to the Society, that I shall not here enlarge on its diagnostic character.

"In the foregoing case, during the course of twelve hours from the first attack (on Tuesday evening) the disease was fully formed. The swelling and erection of the epiglottis; the slow, difficult, and stridulous respiration; without any pain or oppression of the lungs, or the slightest erubescence of the visible internal fauces; the exasperated pulse, and perfect inability of deglutition, marked the disease as cynanche laryngea.

"The copious depletion of blood, already detailed, to the amount of 66 ounces, (which was uniformly buffed and closely cupped,) together with the extravasation of 36 leeches, reduced the general fever, without affording any corresponding local relief. It was, at that juncture, a question whether bronchotomy should be resorted to; and the measure was decided upon, from a consideration of the age and delicate constitution of the patient; and from a persuasion, that time only could reduce the inflammatory tumefaction of the diseased parts. Meanwhile it became essential to facilitate the function of respiration; and to relieve the bowels, and supply nutriment by the rectum. But the nocturnal exacerbations of fever, (unattended however by any observable rigors,) greatly discouraged the hope of final relief.

"On dissection, these phænomena appeared to be accounted for, by the abscess established, and the continual formation of fresh purulent matter.

"It was very evident, both from the symptoms during life, and from the appearances on dissection, that the inflammatory stage of the disorder had entirely subsided some time previous to the dissolution of the patient. So entirely, in truth, had the urgent symptoms ceased, that, twenty-four hours before his death, his medical attendants had formed better hopes of the event of his disorder, than they had ventured to indulge at any preceding period. Respiration and deglutition were restored to their natural facility; the fever, though not subdued, had considerably abated; and the animal spirits, and countenance of the patient, were such as to encourage the hope of ultimate recovery. In the course of the following night and morning, the febrile exacerbation recurred with more violence and more alarming symptoms than ever; delirium, stupor, and at length death supervened, by the process common to febrile exhaustion.

"I have already stated, that the patient had a severe attack of a similar, and not improbably of the same disorder, eleven years ago. Sir Gilbert Blane has obligingly informed me, that 'he was called to a medical attendance on the subject of this case, in London, in February 1802, when the patient, after labouring under catarrh for ten days, was seized with fits of strangulation, which continued, at intervals, for a week, during which period he was thrice bled. The blood was buffy, but not cupped, on the two first occasions; on the third, the buff was absent, and the complaint then assumed a form, so purely

 spasmodic, spasmodic, that opium and assafœtida were given with evident relief.' I have since been informed, that twenty years ago, whilst he was on a journey in Ireland, he was similarly affected, in so severe a degree, that medical aid was summoned from Dublin on the occasion; and that a long time elapsed before he recovered from the severity of the disorder.

"On the late occasion, the unfortunate resolution which he took, of rising from his bed during profuse perspiration, (excited by doses of James's powder,) and of occupying many hours in dictating aloud, conspired at once to expose him to a chill over the surface of his body, and to excite preternatural irritation in the larynx. The predisposition to the specific disorder of cynanche laryngea was not only hereditary, but clearly marked in the individual constitution.

"From the foregoing recital, I think, may be inferred, the expediency of resorting early to the operation of bronchotomy, before the lungs are thrown into a disordered condition; and before the general powers of life are exhausted by the laborious and imperfect exertion of this vital organ. Had not the secret abscess been formed, in the case before us, the general fever would, in all probability, have subsided with the decline of the local inflammation; whilst the intervening restoration of the powers of breathing and deglutition might have been effectual in preserving the life of the patient."

XVI. *Case of Extravasation of Bile into the Cavity of the Abdomen, from Rupture of the Liver or Gall Bladder.* By Mr. FRYER, Surgeon, of Stamford.

This is an extraordinary case, and the patient appears to have had a narrow escape. The subject of it, a boy aged thirteen, received a violent blow from one of the shafts of a cart, on the region of the liver, which was succeeded by pain, and frequent vomiting of bilious matter. He complained of great sinking, and coldness of the extremities, and his pulse was weak, small, and fluttering. A surgeon was sent for, who ordered the abdomen to be fomented; and, as the patient's stomach rejected every thing, he directed purging clysters to be occasionally thrown up. On the third day, symptoms of inflammation coming on, Mr. Cooper, who we believe was Mr. Fryer's partner, was requested to see the child, whom he found then "labouring under considerable pain about the region of the liver; there was great tension of the abdomen, which was extremely sore to the touch, and his vomiting continued as frequent as at first, his stomach rejecting both food and medicine. The pulse was very quick, small, and weak; the skin hot and dry; the tongue much furred; the urine high-coloured; and he complained of some difficulty of breathing, and of great thirst." It is very obvious that with such symptoms, after an accident of the nature above described, the delay of a very few hours more would have rendered the only remedy which occurred to to us on reading the case, likely to afford relief, ineffectual. We hardly need mention bleeding, which Mr. Cooper ordered to the extent of *eight ounces*. The fomentations were continued, and a few grains of calomel were ordered to be given every hour till proper evacuations, which the glysters had failed in bringing down, should be obtained. Afterwards the effervescing mixture, with ten drops of laudanum, every four hours. The following day he was much better, having had some motions; but his sickness still continuing, he was ordered one grain of opium every four hours. Mr. Cooper saw him again in eight days: the day preceding his visit, the boy had complained of great increase of pain, accompanied by vomiting, which was somewhat relieved by a blister. Mr. C. found him completely jaundiced: his stools were white; the swelling and tension of the abdomen were much diminished, and the sickness and thirst abated. The same treatment was continued. Two days afterwards the swelling of the abdomen was increased, and a fluctuation perceptible. In eight days more (twenty-one from the accident) the abdomen was considerably distended with fluid. The patient did not complain of much pain, had the facies Hippocratica, and appeared to be sinking fast. He was now tapped, and thirteen pints of what appeared to be *pure bile* were evacuated. He bore the operation well, but did not seem to derive much benefit from it. The operation was repeated in twelve days, and fifteen pints of a similar fluid were drawn off. "He bore the tapping better than before, experienced great relief, and, though he was still much reduced, was evidently better. In nine days more, the tapping was repeated, and thirteen pints of a similar hue evacuated with like advantage." The operation was once more performed after an interval of about nineteen days, when only six pints were drawn off "A purging had come on about a week before. His stools were of a natural colour; he was much improved in his looks, and his appetite and spirits were good." He continued gaining health and strength, and is now a stout young man.

XVIII. *Some Observations on the Use of Opium in Uterine Hæmorrhagy.* By Mr. STEWART, Lecturer on Midwifery, &c.

The author has detailed two cases to illustrate the utility of opium in uterine hæmorrhage. In the first, in which the patient was reduced almost to extremity, in consequence of a month's flooding, the discharge of blood averaging a pint daily, he gave, at seven o'clock, eighty drops of laudanum:

this producing no sensible effect in twenty minutes, one hundred and twenty drops more were given, which in ten minutes was followed by drowsiness, and a remission of the vomiting and tremors.

"At eight o'clock (Mr. Stewart continues) the hand was introduced into the vagina, the os uteri gradually dilated, the placenta detached at one side, the membranes ruptured, and, the hand being carried forward, the child's feet were grasped, and brought into the vagina; the vomiting and restlessness again recurring, eighty drops of laudanum were given, which produced composure and a permanent cessation of the vomiting.

"The fœtus, which appeared to be of the seventh month, was easily extracted.

"The hand was introduced immediately afterwards, and the uterus instantly contracted, separating the placenta, and forcing it into the vagina, from whence it was gradually extracted.

"At nine o'clock fifty drops of laudanum were given, and at short intervals she took some gruel and brandy.

"At ten o'clock I left her, having ordered a draught containing sixty drops of laudanum, to be given at two o'clock next morning.

"At nine o'clock the following morning, I found she had taken her medicine, had slept two hours, and said she had no complaint; her pulse, which was one hundred and thirty, was very weak, and intermitted.

"I ordered her to take fifty drops of laudanum, some beef tea at very short intervals, and occasionally some gruel and brandy.

"In the evening she was doing well, and her pulse was the same as in the morning. She took sixty drops of laudanum at bed-time.

"The next day in the morning her pulse was one hundred and twenty, weak and intermitting; she had passed a comfortable night, and felt in every respect easy. She was ordered forty drops of laudanum, and the beef tea, brandy, and gruel, were continued. At night I found her the same as in the morning, and ordered her fifty drops of laudanum at bed-time.

"On the following day her pulse was diminished in frequency, was stronger and more regular, and she said she had passed a good night.

"As she had no stool for four days, she was ordered an ounce of castor oil, which operated in the evening; the beef-tea was continued, and she took thirty drops of laudanum at bed-time.

"The two succeeding nights she took thirty drops of laudanum.

"From this period she rapidly advanced to a state of convalescence without the occurrence of one untoward symptom; and in fourteen days from the time I first saw her, she was able to engage in the management of her family."

In the second case, the patient swallowed one ounce of laudanum between the hours of eleven in the morning and seven in the evening of the same day; and continued to take very very large doses of it afterwards, with, it appears from the statements before us, beneficial effects.

The author recommends the following rules to be observed where it is thought expedient to administer opium in uterine hæmorrhage:

"First. Opium ought not to be exhibited, till we have determined that the natural efforts are insufficient to expel the child.

"Secondly. When we have determined upon delivering the patient by turning the child, a large opiate, not less than eighty drops of the tincture, or four grains of solid opium, ought always to be given; and, if the circumstances of the case will permit, we should wait twenty minutes if the tincture, or half an hour if the solid opium is given, before the hand is introduced.

"Thirdly. As often as symptoms of general irritability, or great debility supervene, the opiate ought to be repeated, and the dose increased according to the urgency of the symptoms.

"Fourthly. If the general system has suffered severely from the effects of the hæmorrhagy, the opiate must not be diminished in quantity, or suddenly remitted, although symptoms of irritability be not present."

XIX. *Observations on the Vascular Appearance in the Human Stomach, which is frequently mistaken for Inflammation of that Organ.* By John Yelloly, M.D. Physician to the London Hospital.

Some important facts are stated in this essay, the whole of which merits the deepest attention from practitioners; but our limits forbid our entering at present upon the subjects which Dr. Yelloly has treated with considerable labour and great ability.

(To be continued.)

---

*Des Maladies de la Vessie et du Méat Urinaire, chez les Personnes avancées en Age.* Par M. Nauche, &c. &c.

In a former Number* we took notice of this Treatise on the Diseases of the Bladder, which we were then prevented from completing, but, as the subject is very important, we will now resume the analysis.

*Varicose Veins in the Urethra or Bladder.*—This cause of impediment to the passage of the urine, although noticed by authors, is not usually referred to by surgeons in their practice. We think, however, that it more frequently produces hæmaturia, than retention of urine, or any other symptom. Our author refers us to several writers for an ac-

* February last.

count of the complaint, but, on turning to Morgagni, we find he rather quotes the Sepulchretum Anatomicum, than speaks from his own anatomical observation. But there is no doubt that the veins in the vicinity of the prostate gland will enlarge and burst in many cases where that gland is diseased, and a considerable hæmorrhage is often the consequence, which it is very difficult to restrain, and being a consequence of the diseased state of the gland, can as little be cured as the disease itself. M. Nauche remarks, that the inhabitants of warm climates are peculiarly subject to this affection—"qui ont abusé d'elles-memes, des plaisirs veneriens et des boissons spiritueuses."

The means of cure pointed out are few and inadequate, but they are as good as the case will admit of. It is impossible to destroy the varices. Rest, the horizontal position, aperients, diluent liquors, and the application of leeches, are the principal remedies which are directed. The bougie or catheter should not be used without absolute occasion, although, when there is retention of urine, M. Nauche says, the elastic catheter may be retained a month or six weeks in the bladder, to repress the enlarged veins, which advice we believe he will hardly get many of his patients to pursue.

*Fungous Excrescences of the Bladder, and Caruncles in the Urethra.*—These tumours may arise in any part of the bladder. They are of the size of an olive or larger, sometimes firm and tuberculated, at other times soft and sanguineous, adhering to the bladder. They produce difficulty and frequency, or sometimes retention, of urine, and all the common symptoms of stone, for which disease it is not unfrequently taken; and even the sound will often lead an inexperienced surgeon to suspect the existence of a calculus, so delusive is the perception communicated by it. The disease is rare in young people, but not unfrequent in those of advanced age.

The excrescence in the urethra called caruncle, has met with the fate of almost every subject in medicine or surgery. It has been considered as the most frequent cause of urinary complaints, and gave rise to all the quackery of medicated bougies; and it has afterwards been denied ever to take place in any instance. The truth seems to be equally removed from each of these conclusions. The analogy which exists between the membrane of the urethra and those of the nostrils, vagina, &c. is sufficient to evince the possibility of these excrescences on the former membrane, while the testimony of credible authors, and the still more positive

 evidence evidence of preparations, show that it is far from being an uncommon occurrence. In the treatment, nothing peculiar is recommended by the author distinct from the treatment of stricture of the urethra.

*Cancer of the Bladder or Urethra.*—The author observes that the bladder is seldom originally the seat of cancer, the disease being communicated from the uterus or the rectum. We have, however, witnessed the complaint in a female, beginning in the bladder and proceeding till it finally destroyed the patient, without either the uterus or the rectum being in the least diseased. The glans penis and extremity of the urethra have not uncommonly been affected with it. There seems to be some confusion in the ideas of the author concerning the nature and origin of cancer in general, in the following passage:

"Lorsque le cancer affecte l'urètre, on cherche à remonter à la cause qui l'a déterminé. Si c'est le vice syphilitique, on administre un traitement approprié; si la maladie est due à une fistule urinaire, on tâche de la faire cesser.* On administre aussi, à l'intérieur, les extraits de ciguë, de cynoglosse; on fait des bains locaux avec leurs décoctions, en les unissant aux mucilagineux, pour qu'ils ne soient pas irritans et n'agissant pas comme styptiques, ce que j'ai vu arriver plusieurs fois. L'on retire aussi quelqu' avantage des bains locaux avec les décoctions, ou les sucs de douce-amére, de laitue sauvage de morelle. D'autres fois, on n'éprouve de soulagement que des bains locaux mucilagineux et calmans.

"Les caustiques partiels sont dangereux; ils ne font que hâter la dégénérescence cancéreuse. Cependant, lorsque la maladie ne fait que commencer, qu'elle n'intéresse qu'une petite surface du gland, l'on peut, selon M. Dubois, en opérer la guérison par l'application d'une pâte arsénicale qui détruit avec célérité tout la surface affectée.

"La maladie occupe-t-elle tout le gland, il faut se hâter d'en faire l'extirpation, avant qu'elle se soit propagée jusqu'à la racine du membre viril, ou qu'elle ait produit l'engorgement des glandes de l'aine, ce qui rendrait l'opération infructueuse.

"Pour faire cette extirpation, on saisit le membre viril avec la main gauche, ayant la précaution de retirer les tégumens vers le gland, et l'on retranche la partie malade avec un bistouri.

"Après avoir fait la ligature des vaisseaux qui donnent du sang, on introduit une sonde dans la vessie, pour que l'urètre et les corps caverneux ne se retirent pas du côté du pubis, et que l'ouverture du canal ne soit pas oblitérée par la peau qui se replie sur elle même, et par la cicatrisation de la plaie.

"Si les corps caverneux, malgré la ligature des vaisseaux, fournissaient trop de sang, on arrêterait l'hémorragie au moyen de la

"* *Voyez* des Fistules urinaires."

compression compression et des styptiques. La plaie doit ensuite être traitée avec un pansement ordinaire."

We do not deny that a venereal sore may sometimes become cancerous, unusual as the occurrence is; but, when the author goes on to designate the callosities which surround a urinary fistula by the same name, it will account for the cures of cancer which are often talked of, but which we fear have never taken place where the disease has been of the true carcinomatous structure.

We beg leave to make one more remark on this passage of the author on the amputation of the penis. The use of the sound into the bladder after the operation, we believe is not only unnecessary, but often injurious to the patient.

The chapter on urinary calculi in the bladder and urethra contains many very useful remarks, but, as their utility is more conspicuous than their novelty, we shall not particularly notice them. The smaller species of calculus which is so frequently found in the diseased prostate gland, is well described in the following chapter; and it will be remarked that the opposite lobes of the prostate gland are very differently affected with disease, in the case here described, which, whatever be its cause, we have often remarked in similar cases, one side or lobe often being considerably enlarged, while the other is but little altered.

" *Des calculs de la prostate.*—Il peut se former dans la substance même de la prostate de petits calculs d'une nature particulière, bien observés par Morgagni,* et que j'ai eu occasion de rencontrer moi-même.

" Ces calculs sont logés dans des sinus ou dans une sorte de canal excréteur, creusé dans la prostate ou même à l'embouchure des conduits éjaculateurs. Quelquefois on n'en trouve que deux ou trois; le plus souvent il y en a un grand nombre. Tantôt ils sont trèspetits, du volume d'un grain de millet; tantôt, aussi gros qu'une cerise: la plupart sont globuleux, transparens, d'un rouge de grenat ou d'un jaune safrané. On a dit qu'ils étaient de même nature que les calculs urinaires; il est vrai que ces derniers calculs peuvent s'arrêter ou même se former dans quelque cavité ou sinuosité de la membrane interne de l'urètre, à l'endroit de la prostate; mais les calculs qui se forment dans la propre substance de cette glande sont de toute autre nature, et l'analyse chimique n'en a pas été faite. J'en ai envoyé une petite quantité à MM. Fourcroy et Vauquelin en les invitant à la faire.

" Ces calculs ne proviennent point d'une communication du sphincter de la vessie ou des environs avec la prostate, comme l'ont avancé quelques auteurs; ils sont étrangers aux vices de l'urine: les

* *Voyez* De sed. morb. calculi in prostatis, t. 2, p. 422.

causes causes qui les déterminent sont tout-à-fait inconnues. Il produisent ordinairement une tuméfaction lente de la prostate, et par suite son inflammation chronique, à moins que cette inflammation ne donne lieu elle-même à la naissance de ces calculs.

" J'ai fait avec M. Mareschal, il y a peu de mois, l'examen de la vessie d'un homme de soixante ans, mort à la suite d'une rétention d'urine; la vessie était en bon état, la prostate très-gonflée, divisée en deux lobes; l'un, du côté droit, dur, rougeâtre, enflammé; l'autre, du côté gauche, blanchâtre, très dense, avec quelques points de suppuration, contenait dans son intérieur une grande quantité de petits calculs, tandis que l'autre lobe n'en contenait aucun.

" Quelquefois ces calculs se fraient un passage jusque dans l'urètre, et sont ensuite rendus avec l'urine; les symptomes qu'ils ont occasionnés disparaissent, et les malades sont rendus à la santé.

" Je connais un homme de lettres, qui, après avoir éprouvé pendant près de quatre mois des doleurs, des difficultés d'uriner, rendit quelques-uns de ces calculs et se trouva délivré de tous ces accidens. Depuis tres ans, l'urine a repris son libre cours.

" Les calculs de la prostate sont peu dangereux par eux-mêmes, mais bien par le gonflement qu'ils déterminent.

" On doit se borner dans leur traitement à calmer les symptômes inflammatoires auxquels ils donnent lieu, et, si le cours de l'urine est intercepté, dilater au moyen des bougies la portion prostatique de l'urètre; attendre que la nature procure elle-même l'évacuation de ces calculs, et employer ensuite les moyens indiqués contre l'engorgement de la prostate.* Si cependant ces calculs faisaient saillie au périnée, ce qui doit être extrêmement rare, on en pourrait faire l'extraction, en faisant une incision sur la tumeur à l'endroit de cette glande."

The presence of worms of different species in the bladder, forms the subject of the following chapter. No distinct diagnosis is or can be given of this affection, but the appearance of them when evacuated; and even this is subject to delusion and imposition. The only means recommended by the author is the use of mercurial injection into the bladder.

Whatever has been thought necessary on so important a subject as the induration of the prostate, is comprised in less than *three pages*, and it is not surprising. But M. Nauche has condensed almost all the useful information we possess on the subject in this small compass; even the pretended discovery of Sir Everard Home is very clearly described and anticipated. We shall extract the whole chapter, making our protest at the same time against the idea that the disease is the consequence of lues venerea. We have, however, before commented on the indistinct notions of the generality of French surgeons on this subject.

---

* *Voyez* de l'Inflammation de la prostate et de son endurcissement.

"De

" *De l'endurcissement de la prostate.*—Sans avoir éprouvé aucune inflammation apparente, la glande prostate est sujette à augmenter de volume, à s'engorger et à acquérir par degrés un endurcissement squirreux.

" Cet endurcissement se reconnait aux douleurs et aux difficultés d'uriner qu'il occasionne. Le jet du liquide diminue insensiblement et finit par disparaître; en portant profondément un doigt dans le rectum, on trouve cette glande tuméfiée et indolente.

" L'introduction d'une sonde dans l'urètre est facile jusqu'à la glande, et éprouve ensuite la plus grande difficulté.

" Cet engorgement, très-fréquent dans le moyen âge, est rarement guéri dans un âge avancé: il est ordinairement la suite d'une inflammation syphilitique de la prostate; quelquefois il survient, sans être précédé de symptômes inflammatoires, et paraît déterminé par la présence de petits calculs développés dans sa substance.* La prostate acquiert peu à peu beaucoup de volume, devient molle, fongueuse, sans éprouver d'altérations sensibles dans son tissu. Dans quelques cas, il n'y a d'affecté que la partie de la glande à laquelle on donne le nom de luette vésicale.

" Cette luette, suivant, M. Sabatier, peut former une tumeur ronde, d'un demi-pouce de diamètre, portée sur un pédicule étroit. Elle s'oppose au passage de l'urine en se plaçant à l'entrée du col de la vessie, sur lequel elle est entrainée par le flot du liquide.

" L'engorgement de la prostate est très-fâcheux, à raison de la difficulté que l'on éprouve à en obtenir la guérison, de la rétention d'urine qui en est la suite, et de l'impossibilité ou l'on est souvent de sonder le malade, ce qui peut nécessiter la ponction de la vessie, laquelle doit être pratiquée alors au-dessus du pubis.

" Cet engorgement reconnu, l'on examine s'il n'y a pas de symptômes de la maladie syphilitique, et dans ce cas l'on fait subir un traitement approprié; on retire souvent de bons effets des frictions au périnée avec l'onguent néapolitain double, dans l'intérieur même du rectum, à l'endroit ou la glande prostate fait saillie; les douches ascendantes, dirigées sur le fondement, sont aussi parfois très-utiles; mais ce sont principalement les bougies et les sondes de gomme élastique qui produisent les meilleurs eff[illegible]ts. Leur introduction, avec des grosseurs graduées, dilate peu à peu la portion de l'urètre située dans l'epaisseur de la glande, et facilite l'écoulement de l'urine, dont la suspension est l'accident le plus dangereux de l'engorgement.

" Il est bon de recourir aux bougies dès les premiers tems de la maladie, et de revenir à leur usage dès qu'on s'aperçoit d'une nouvelle diminution dans le calibre du canal, de crainte que ce dernier ne s'obstrue entiérement, ce qui mettrait la vie des malades en danger."

The chapter on stricture of the urethra contains nothing either new or remarkable, but the unusual candour and ingenuousness of the author in allowing the constant and ne-

* *Voyez* des Calculs de la prostate.

cessary cessary disposition of strictures to return after they have once affected the urethra. In this we believe he is almost single among the many writers on the subject: there are no boasts of permanent cure by caustic or other means, but a frank avowal of what is really the truth of the case. "It is a law," says the author, "common to every canal destined to give passage to fluids, that, when they have once experienced an alteration of structure, it is almost (he might have said quite) impossible to restore them to their primitive state. Thus, persons who have had a fistula lachrymalis, or an involuntary flow of tears, from an ulcer in the inner angle of the eye, and obstruction of the lachrymal duct, are always subject and predisposed to a return of the complaint, in whatever mode they may have been cured.

The remaining chapters of this little manual or treatise are occupied on the use of the bougie and other modes of relieving strictures, and their consequences, such as fistula in perinæo, suppression of urine, &c. which, as there is no peculiarity in the pathology or treatment, we shall not pursue in detail, but conclude our analysis with observing, that, with some allowance for the vague notions of his countrymen on syphilis, it may be in general considered as a concise and good abridgment of the nature and treatment of the diseases of the bladder and urethra,—not only those of old age, as its title imports, but those also which are incident to the more early periods of life.

---

*Observations on those Diseases of Females which are attended by Discharges;* by CHARLES MANSFIELD CLARKE, Member of the Royal College of Surgeons, &c. Longman and Co. 8vo. pp. 304.

WE congratulate the profession upon the appearance of a publication on a subject of great practical importance, from the pen of Mr. C. CLARKE. In the treatment of diseases of Females, few have had more extensive opportunities than this gentleman, and still fewer possess an equal ability to render that experience profitable to mankind. Too often are we doomed to fret over the pages of the visionary theorist, and but rarely called upon to record the sentiments of the real practitioner: few, indeed, engaged in the active duties of their profession, feel sufficient interest or philanthropy to take upon them the less profitable task of the writer—an office consigned to the mere adventurer, who hopes to be and frequently is successful, in proportion to the number of lies he can tell. The object of Mr. Clarke, in the present publication, is to make some arrangement in the sexual diseases of females; and to show that diseases having some symptoms in common, are nevertheless very dissimilar in character, and require very different treatment: it occurred to him that some are attended by no discharge from the vagina; others, on the contrary, never occur without it, and the nature of the discharge varies. Here then appeared a ground of classification, the utility of which is illustrated by the following supposition:—A practitioner is not quite sure whether a tumour in the vagina is a soft polypus or a cauliflower excrescence; if he knows that one of those diseases is attended by a mucous discharge, and the other by a discharge of water, he has nothing to do but to inquire into this circumstance, and the question is immediately solved. The entire work will consist of a series of which the present volume is but the first part, and is devoted to the consideration of *mucous discharges*. The purulent watery and white opaque discharges will be considered in the remaining parts.

The author observes, that "The discharges from the parts of generation come away from the os externum; but they spring from various sources, and are of different kinds. The parts from which these secretions arise, are:

1. The internal surface of the uterus and of the fallopian tubes.
2. The inner membrane of the vagina.
3. The lacunæ about the os externum.
4. The mucous membrane of the urethra.*

These will be separately considered.

1. The secretions from the uterus. These are:

α. The menstruous secretion.

β. The secretion from the mucous membrane of the uterus, which extends to the cavities of the fallopian tubes.

γ. The secretion from the glands in the neighbourhood of the cervix of the uterus."

(To be continued.)

* "These are the surfaces from which the natural secretions arise: but discharges from the os externum may originate from the surfaces of newly-formed tumours, as the cauliflower excrescence; or they may be the contents of cysts of hydatids."

